# How the Germans Treat Wounded Prisoners: The Testimony of a French Doctor

**Published:** 1915-10-02

**Authors:** 


### HOW THE GERMANS TREAT WOUNDED PRISONERS.

### The Testimony of a French Doctor.

On different occasions the most revolting accounts of the behaviour of German soldiers, both officers and men, in France and Belgium have been made public, and the report of a Commission which carefully investigated a good many of these cases was a terribly convincing indictment. As far as judicial and expert investigation of lay testimony can go, it is absolutely certain that on very many occasions the behaviour of the Germans has been so brutal, so bestial, that no words of condemnation are strong enough to express the feelings with which civilised nations regard them. Not only in that report, but on many other occasions, there have been forthcoming corroboration and confirmation of the horrors which German occupation inflicts upon women and children, old men and maidens, and defenceless captives, wounded or unwounded. Not on isolated occasions, either, have the enemy fired deliberately upon the Red Cross and on buildings protected by it, and have violated in letter and in spirit practically every canon of the Geneva and Hague Conventions. Such stories have become so common that there is a danger of public feeling getting inured to them, and of the fine edge of popular indignation being blunted by the mere frequency with which it is called forth. One of the latest examples of that inherent inhumanity which the German soldier seems unable to exorcise from his nature is furnished by the narrative of a French military surgeon, recently returned from eleven months' captivity among the Huns, fragments of whose experiences have been reproduced from the *Gaulois* in the London *Standard*.

### Firing on the Red Cross.

First of all, on the battlefield where he was taken prisoner, the German artillery shelled his aid-post. Certainly it is not always easy for gunners to see and spare the Red Cross; and it must always be somewhat in doubt whether any given instance of a shelled hospital is due to deliberate intention. But the Germans have done it so regularly and so persistently that there is no reasonable doubt that their gunners purposely shell hospitals when well aware of their nature. Then came the infantry, who fired repeatedly at the doctor and his stretcher party, wounding some of them. After that, seeing red apparently, the Württemberg troops committed horrible excesses. "What was to become of us, in presence of these madmen who yelled as they rushed in upon us, using the butt-ends of their rifles without mercy, although we showed them our Red Cross badges?" asks the doctor. The prisoners were, as usual, despoiled of all their possessions by their captors, a violation of the laws of war in the case of combatant prisoners, and doubly prohibited in that of Red Cross workers. Threatened with execution, and prodded in the back with a bayonet, the French doctor was led away along a road where wounded Frenchmen, imploring their savage captors for water, were being shot in cold blood as they lay. Not even the corpses were spared the satanical fury of the drunken bands: heads were hacked from bodies and blood-lust ran riot everywhere.

For several days the doctor was kept in a Belgian frontier town to look after the French wounded, to whom food, dressings, and even water were refused. "Dying of hunger and thirst, we were reduced to looking for the crumbs of biscuits that the Germans had overlooked in rummaging the knapsacks of their victims."

### Inside the German Hospitals.

Eventually this eye-witness reached a hospital where on the whole he and the wounded were "relatively" well treated. Many accounts by independent witnesses give us the impression that inside the German hospitals the worst excesses of the soldiers and the populace are no longer allowed or encouraged; though there have been places and occasions when this limited testimonial cannot be given. At any rate there was no gross brutality in this particular hospital, possibly because the nurses were Luxemburgers. But before reaching the hospital the doctor was "pitilessly refused" some anti-tetanus serum by the German surgeons. Eventually he was sent to a prison camp, where systematised and persistent ill-treatment and starvation of the sort which seems to be the recognised rule in Germany were meted out.

There is, of course, nothing in this narrative worse than—nay, nothing half so bad as—many of the well-attested incidents in which German cruelty has been exposed to the world already. It is only one small item in the dark catalogue of misdeeds which have made the name of Germany stink in the nostrils of the whole non-German world. But we have thought it worth while to draw attention to this testimony of one of our French colleagues lest righteous anger die down in our midst, and as one more incident of the abrogation of the Geneva Convention which the German Army practises.

